# Interfacial effects on leakage currents in Cu/α-cristobalite/Cu junctions

**DOI:** 10.1038/s41598-020-62356-6

**Published:** 2020-03-24

**Authors:** Kuan-Bo Lin, Yen-Hsun Su, Chao-Cheng Kaun

**Affiliations:** 1 Research Center for Applied Sciences, Academia Sinica Taipei, 11529 Taiwan; 2 Department of Materials Science and Engineering, National Cheng Kung University, Tainan, 70101 Taiwan; 3 Department of Physics, National Tsing Hua University, Hsinchu, 30013 Taiwan

**Keywords:** Nanoscience and technology, Physics

## Abstract

As the miniaturization trend of integrated circuit continues, the leakage currents flow through the dielectric films insulating the interconnects become a critical issue. However, quantum transport through the mainstream on-chip interfaces between interconnects and dielectrics has not been addressed from first principles yet. Here, using first-principles calculations based on density functional theory and nonequilibrium Green’s function formalism, we investigate the interfacial-dependent leakage currents in the Cu/α-cristobalite/Cu junctions. Our results show that the oxygen-rich interfaces form the lowest-leakage-current junction under small bias voltages, followed by the silicon-rich and oxygen-poor ones. This feature is attributed to their transmission spectra, related to their density of states and charge distributions. However, the oxygen-poor interfacial junction may conversely have a better dielectric strength than others, as its transmission gap, from −2.8 to 3.5 eV, is more symmetry respect to the Fermi level than others.

## Introduction

Whereas transistors evolve towards their physical limits^1,2^, the dielectrics^3–8^ separating the copper interconnects^9,10^ follow the miniaturization trend, and the leakage currents flow through them become a critical issue. Although high-k material films can reduce the leakage current, the company parasitic resistance-capacitance loop in between such films and interconnects delay the signal propagation^11,12^. The silica compound thus still constructs the mainstream on-chip insulation films and remain being a research focus of nanoelectronics. For example, first-principles calculations were performed to address electronic and tunneling properties in silica layers^13–15^, and the electron-energy-loss spectroscopy was exploited to resolve the chemical composition and electronic structure of the Si/SiO_2_ interface^16^, suggesting that the insulation limit thickness for an ideal silica oxide layer is ~0.7 nm (four Si planes across).

Nature adhesion ability between Cu atom and oxide surfaces is not as good as Al^9,17^, but, due to the convenience of planarization and the 36% of lower resistivity^12^, the mainstream on-chip high-speed propagation interconnects are still dominated by copper local wires. Nevertheless, the properties of the Cu/SiO_2_ interface are rarely studied computationally^18,19^, showing that the interfacial states are sensitive to the oxygen density on the Cu/SiO_2_ interface, since the highest adhesion energy and bonding strength happen on Cu (001) and α-cristobalite (001) slabs interface. However, first-principles study on quantum transport through such kind of dielectric/metal interface is still limited.

In this work, we study the interfacial-dependent leakage currents in the Cu/α-cristobalite/Cu junctions, with the insulation limit thickness of silica layers, from first principles. Our results show that the oxygen-rich interfaces form the lowest leakage-current junction, followed by the silicon-rich and oxygen-poor ones, due to the difference in their density of states (DOS) and charge distributions. The interfacial-dependent dielectric strengths are also addressed.

## Results and Discussion

**Figure 1:**
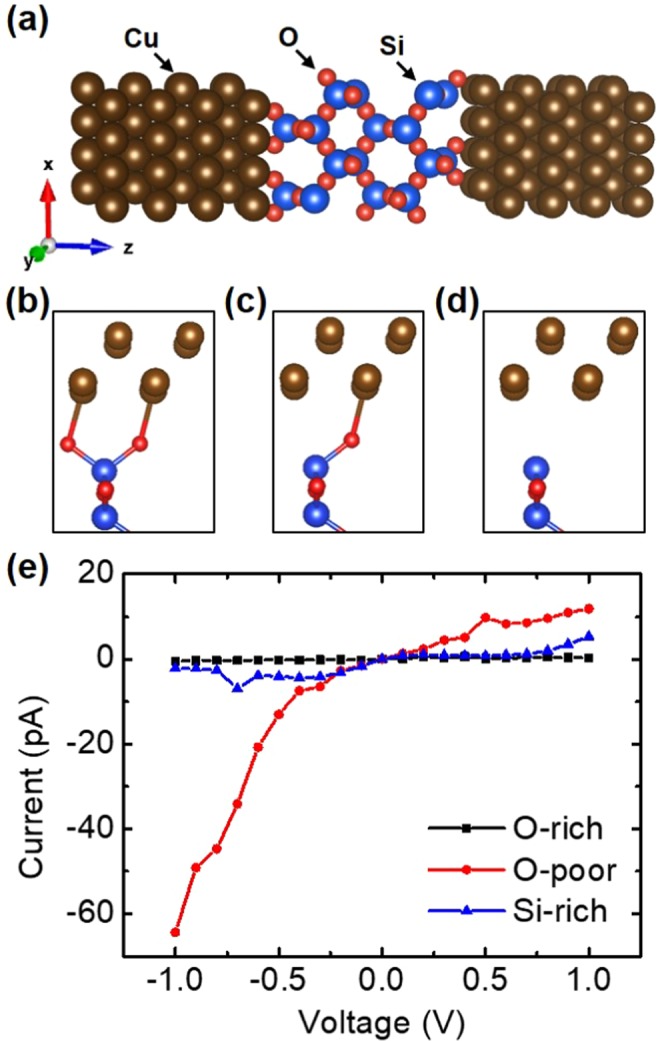
(**a**) Geometric structure of the junction consisted of an α-cristobalite (001) slab in between Cu (001) electrodes, with (**b**) oxygen-rich, (**c**) oxygen-poor, or (**d**) silicon-rich interfaces. (**e**) Current-voltage characteristics of the junctions.

Figure 1a plots the Cu/α-cristobalite/Cu junction consisted of an α-cristobalite (001) slab, four Si layers about 1.6 nm thick, in between Cu (001) electrodes. The SiO_2_-Cu interfaces can be oxygen-rich (100% oxygen), oxygen-poor (50% oxygen), or silicon-rich (0% oxygen) configurations as shown in Fig. 1b–d, respectively, where the oxygen-poor one is formed by removing the half of O atoms from the α-SiO_2_ surface, so that O vacancies are generated in between α-cristobalite (001) slab and Cu electrode, and the silicon-rich interface is constructed by remove all O atoms from the SiO_2_ surface. Figure 1e shows the leakage currents of the junctions under different bias voltages. The current in the oxygen-poor interfacial junction is around seventeen (five) times larger than that in the silicon-rich one at −0.8 (0.8) V, while that in the oxygen-rich interface is almost zero. The worst insulation barrier thus occurs on the oxygen-poor interface and the best one is the oxygen-rich interface.

**Figure 2:**
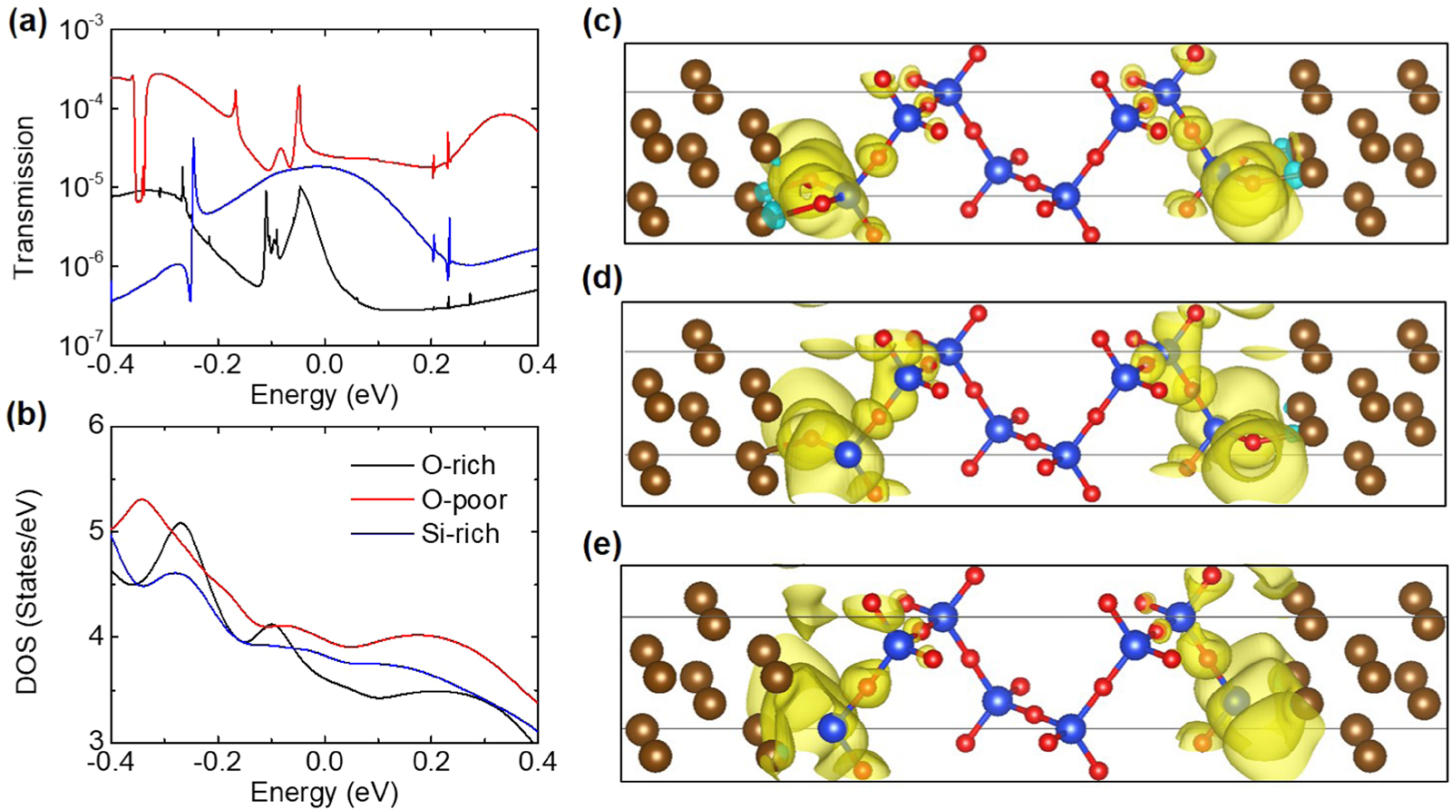
(**a**) Transmission spectra and (**b**) density of states (DOS) of the junctions, with the same color code of Fig. 1e. Local density of states (LDOS) at E_F_ of the junctions with (**c**) oxygen-rich, (**d**) oxygen-poor, and (**e**) silicon-rich interfaces.

Transmission spectra of the junctions with three types of interfaces are shown in Fig. 2a, with the same color code of Fig. 1e. The junctions with oxygen-poor, silicon-rich and oxygen-rich interfaces have the high, middle and low transmission coefficients around the Fermi level (E_F_), respectively. Density of states (DOS) of the junctions are presented in Fig. 2b, which line shapes are similar to their transmission spectra and indicate that they are corresponding. Moreover, the local density of states (LDOS) at E_F_ of the junctions with oxygen-rich, oxygen-poor, and silicon-rich interfaces are depicted in Fig. 2c–e, respectively, where the isosurface is 0.0001 e/Å^3^. The LDOS distribution of the oxygen-rich interface is the most localized, whereas that of the oxygen-poor one is converse. This indicates that the charges tend to accumulate on the vacancy sites of the oxygen-poor interface and penetrate into the α-cristobalite (001) slab, leading to the poorest insulation quality of the oxygen-poor interface.

**Figure 3:**
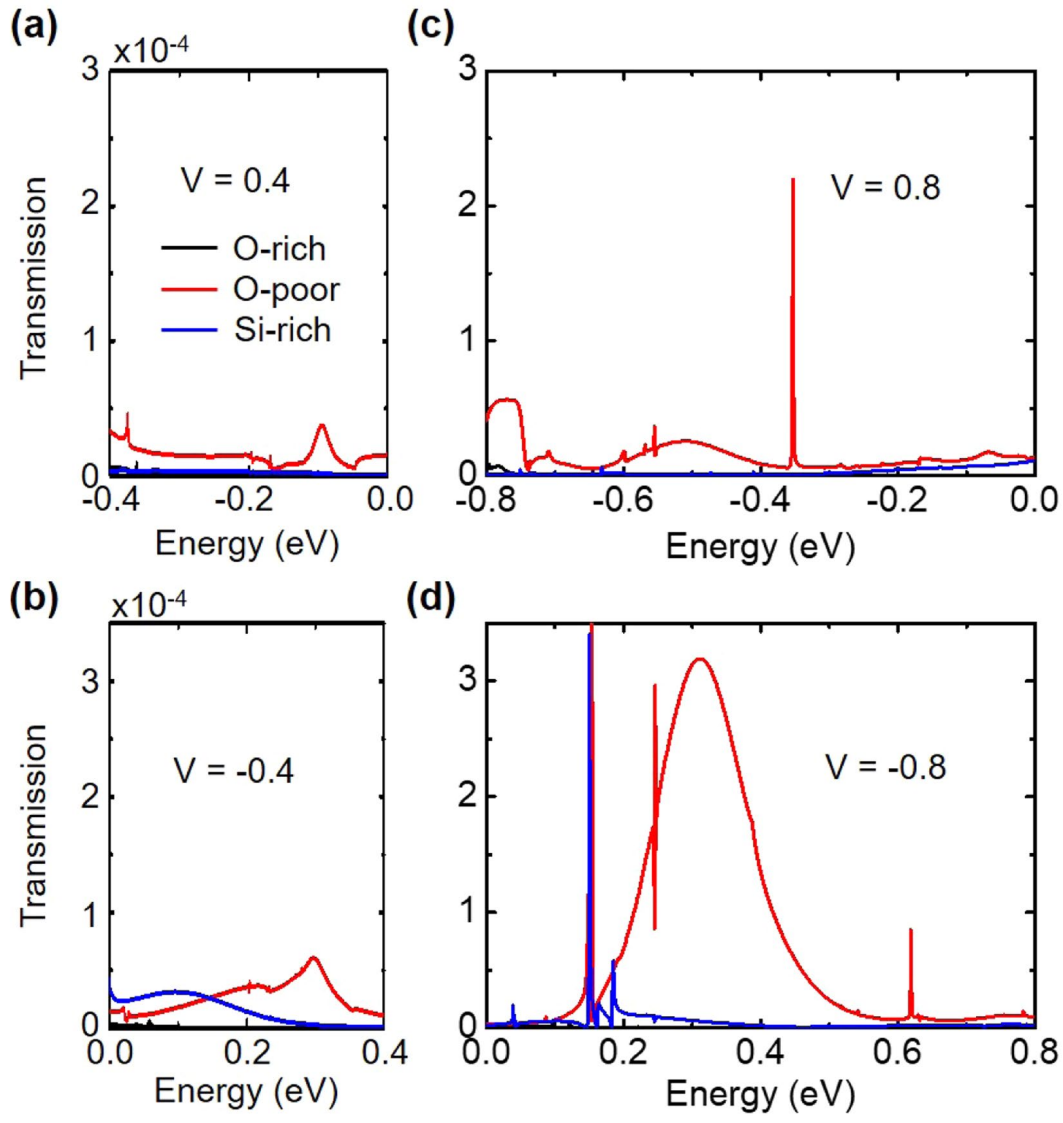
Transmission spectra of the junctions under different bias voltages.

Figure 3(a,b) shows transmission spectra of the junctions with O-poor, Si-rich and O-rich interfaces under bias voltages of 0.4 (−0.4) V. The area under the transmission curve, i.e. the current, of the O-poor system is larger than that of the Si-rich one, in particular at the positive voltage. Figure (3c,d) plots transmission spectra of such the junctions under bias voltages of 0.8 (−0.8) V. The area under the transmission curve of the O-poor system is much larger than that of the Si-rich one at the negative voltage. The areas under the transmission curves of the O-rich system, however, is almost zero in all cases.

**Figure 4:**
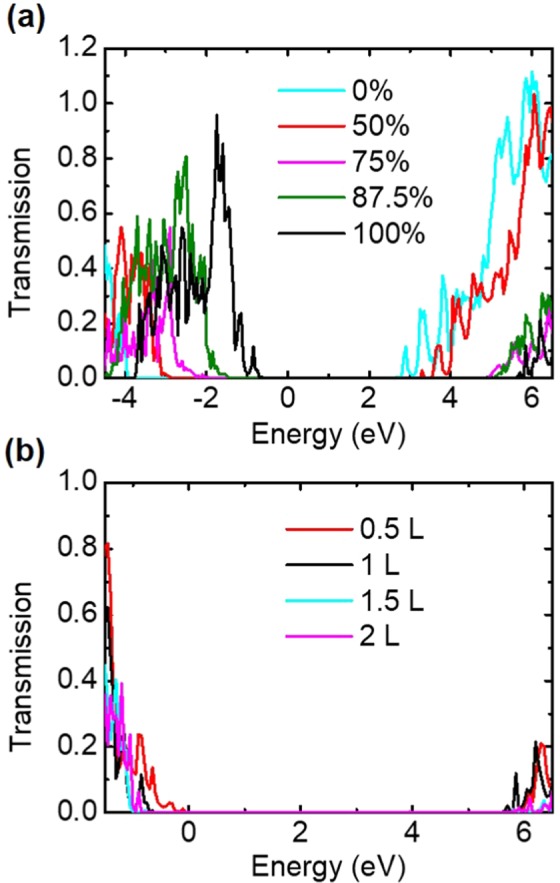
Transmission spectra of the junctions at a wider energy range with (**a**) concentration-dependence and (**b**) thickness-dependence (for the 100% oxygen interface).

To investigate the possible dielectric strength, i.e. the breakdown voltage, transmission spectra of the junctions with different oxygen-concentration interfaces at a wider energy range are shown in Fig. 4a, whereas the current-voltage characteristics of the junctions at such a huge bias is computationally too heavy to achieve. The interfacial O-rich junction shows an asymmetric transmission gap, from −0.8 to 5.7 eV (where the transmission coefficient is 0.01), respect to E_F_, suggesting a breakdown voltage around 1 V. Reducing the interfacial oxygen concentration shifts the asymmetric transmission gap toward lower energies, so that the interfacial Si-rich junction has an asymmetric transmission gap, from −3.9 to 2.6 eV, respect to E_F_, leading to a possible breakdown voltage of −2.6 V. However, the interfacial O-poor junction owns the most symmetric transmission gap, from −2.8 to 3.5 eV, respect to E_F_, offering a breakdown voltage of around 3 V. Therefore, the transmission gap symmetry, i.e. locations of valence and conduction band offsets, can be shifted as the interfacial oxygen concentration is changed, so that the leakage current and the dielectric strength of few-layered α-cristobalite (001) slab can be tuned by engineering the concentration of O atoms on the interfaces. In addition, Fig. 4b shows the transmission spectra of the O-rich junctions with different insulation thicknesses. Whereas thinning the slab decreases the transmission gap, thickening it does not change such gap much.

## Conclusions

We have shown the interfacial effects on leakage currents in Cu/α-SiO_2_/Cu junctions for the oxygen-rich, oxygen-poor, and silicon-rich interfaces. The oxygen-rich interfaces form the lowest leakage-current junction under the bias of ±0.8 V, followed by those junctions with the silicon-rich and oxygen-poor interfaces. This feature comes from the characters of their transmission spectra at equilibrium and under bias. The oxygen-rich interfacial junction has the lowest transmission coefficients, related to its lowest DOS and the most localized interface states. However, for the purpose of a good dielectric strength (the breakdown voltage), the oxygen-poor interfacial junction may play a better role, as its transmission gap, from −2.8 to 3.5 eV, is more symmetry respect to E_F_. These results would help the development of the isolation layers between the Cu electrodes for nanoelectronics.

## Methods

The junction geometries were optimized by using *Siesta*^20^, a code based on density functional theory (DFT). All atoms at each interface (two Cu and two α-cristobalite layers) were allowed to relax. The lattice mismatch of α-cristobalite and Cu primitive cell was 37.7%, so that the Cu (001) unit cells were rotated by 45° to sandwich the α-cristobalite (001) slab and thus construct the supercell (58, 56, and 54 atoms for interfacial O-rich, O-poor, and Si-rich junctions, respectively), reducing the lattice mismatch to less than 1%^18^, (see Supplementary Information). Energy convergence tolerance was *dE* < 10^−4^ eV and maximum residue force tolerance *dF* < 0.04 eV/Å. An energy cutoff was 100 *Ryd*. The quantum transport properties were calculated by using *Nanodcal*^21^, a code based on DFT and nonequilibrium Green’s function formalism. Transport direction was along the z-axis, whereas the x- and y-axis were selected in the periodic boundary condition. Four layers of Cu on each electrode were chosen as the buffer layers. Energy convergence criteria was *dE* < 10^−4^ eV and energy cutoff was 80 *Hartree*. The Monkhorst-Pack *k*-points mesh was selected as *k* = 4 × 4 × 100, corresponding to the real-space grids number *n* = 41 × 41 × 238. The currents were calculated by using Landauer’s formula as expressed,

1 $$I=\frac{2e}{h}{\int }_{-\infty }^{+\infty }dE({f}_{R}-{f}_{L})T(E)$$

where *f*_*R*_ − *f*_*L*_ is Fermi-Dirac occupation function difference between two Cu electrodes and *T*(*E*) is the transmission spectrum. Both calculations used pseudopotentials and the double-ζ plus polarization basis set, within the local density approximation.

When oxygen concentrations differ from 100%, 50% and 0%, disorder effects (oxygen-position-dependent) occur in the tunnel leakage. Such effects can be handled by coherent potential approximation (CPA) by which the disorder average of retarded Green’s function can be carried out analytically^22^, and they have been addressed, for examples, in magnetic tunnel junctions^23^, Cu interconnects^10^, and Si nanotransistor channels^24^. However, disorder effects are neglect in this work.

## Supplementary information

Supplementary information.
