# Supplementary material for: Interfacial effects on leakage currents in Cu/α-cristobalite/Cu junctions
Source: Sci Rep. 2020 Mar 24;10:5303. doi: 10.1038/s41598-020-62356-6 (PMC7093521; doi:10.1038/s41598-020-62356-6)
Supplement: Supplementary file 1 — Supplementary information. [file 41598_2020_62356_MOESM1_ESM.pdf]

Interfacial effects on leakage currents in Cu/α-cristobalite/Cu junctions

Kuan-Bo Lin, Yen-Hsun Su & Chao-Cheng Kaun

Supplementary Information

S1. Atomic coordinates

The lattice structure of Cu is face centered cubic (fcc) and α-cristobalite is tetragonal. Therefore, two Cu (001) electrodes are rotated by 45° in x-y plane to sandwich the α-cristobalite (001) slab. The atomic coordinates of the O-rich (100% oxygen) junction for *Nanodcal* calculations (lead/scattering region/lead) are following.

Atom Types	X	Y	Z
Cu	4.40269560	0.57589944	0.90367540
Cu	1.84671492	0.57589944	0.90367540
Cu	1.84671587	3.13187344	0.90367540
Cu	4.40269560	3.13187344	0.90367540
Cu	0.56872553	1.85388787	2.71102556
Cu	3.12470526	1.85388882	2.71102556
Cu	0.56872553	4.40986283	2.71102556
Cu	3.12470621	4.40986283	2.71102556
Cu	4.40269560	0.57589944	4.51837762
Cu	1.84671492	0.57589944	4.51837762
Cu	1.84671587	3.13187344	4.51837762
Cu	4.40269560	3.13187344	4.51837762
Cu	0.56872553	1.85388787	6.32572778
Cu	3.12470526	1.85388882	6.32572778
Cu	3.12470621	4.40986283	6.32572778
Cu	0.56872553	4.40986283	6.32572778
O	2.65437050	1.32110850	8.22572734
O	0.01937650	1.34095550	8.24826134
Si	1.34490050	1.34212650	9.22682934
O	1.34433950	2.70875250	10.13862234
O	1.36174650	5.41590450	10.16417434
Si	1.34829450	4.05791150	11.06456334
O	5.41316650	4.05522350	11.97906934
O	2.70133750	4.05152750	11.98383234

Si	4.05961850	4.05553350	12.89499134
O	4.06111350	5.41271250	13.80940034
O	4.06709650	2.70124350	13.81234734
Si	4.07486450	1.35012550	14.73298634
O	0.00927350	1.36356450	15.64999034
O	2.72133350	1.35962750	15.65305734
Si	1.36470450	1.34774650	16.56624334
O	1.33643350	2.70797250	17.47881334
O	1.39924650	5.41813650	17.49011534
Si	1.34923350	4.06179050	18.39625734
O	2.72137450	4.06031350	19.28463434
O	0.01131450	4.05881450	19.34637834
Si	4.07698050	4.06326050	20.19534434
O	4.07823450	2.69683550	21.09379434
O	4.07696950	0.00653550	21.09598234
Si	4.07826250	1.38403150	22.07649334
O	2.79833650	1.49975750	23.11519134
O	5.34777750	1.51345650	23.13017434
Cu	4.40269560	0.57589944	24.95222505
Cu	1.84671492	0.57589944	24.95222505
Cu	4.40269560	3.13187344	24.95222505
Cu	1.84671587	3.13187344	24.95222505
Cu	0.56872553	1.85388787	26.75957521
Cu	3.12470526	1.85388882	26.75957521
Cu	0.56872553	4.40986283	26.75957521
Cu	3.12470621	4.40986283	26.75957521
Cu	4.40269560	0.57589944	28.56692537
Cu	1.84671492	0.57589944	28.56692537
Cu	1.84671587	3.13187344	28.56692537
Cu	4.40269560	3.13187344	28.56692537
Cu	0.56872553	1.85388787	30.37427552
Cu	3.12470526	1.85388882	30.37427552
Cu	0.56872553	4.40986283	30.37427552
Cu	3.12470621	4.40986283	30.37427552
Cu	4.40269560	0.57589944	-6.32572492
Cu	1.84671492	0.57589944	-6.32572492
Cu	0.56872553	1.85388787	-4.51837476
Cu	3.12470526	1.85388882	-4.51837476

Cu	1.84671587	3.13187344	-6.32572492
Cu	4.40269560	3.13187344	-6.32572492
Cu	0.56872553	4.40986283	-4.51837476
Cu	3.12470621	4.40986283	-4.51837476
Cu	4.40269560	0.57589944	-2.71102492
Cu	1.84671492	0.57589944	-2.71102492
Cu	0.56872553	1.85388787	-0.90367476
Cu	3.12470526	1.85388882	-0.90367476
Cu	1.84671587	3.13187344	-2.71102492
Cu	4.40269560	3.13187344	-2.71102492
Cu	0.56872553	4.40986283	-0.90367476
Cu	3.12470621	4.40986283	-0.90367476
Cu	4.40269560	0.57589944	32.18167464
Cu	1.84671492	0.57589944	32.18167464
Cu	0.56872553	1.85388787	33.98902504
Cu	3.12470526	1.85388882	33.98902504
Cu	4.40269560	3.13187344	32.18167464
Cu	1.84671587	3.13187344	32.18167464
Cu	0.56872553	4.40986283	33.98902504
Cu	3.12470621	4.40986283	33.98902504
Cu	4.40269560	0.57589944	35.79637464
Cu	1.84671492	0.57589944	35.79637464
Cu	0.56872553	1.85388787	37.60372504
Cu	3.12470526	1.85388882	37.60372504
Cu	4.40269560	3.13187344	35.79637464
Cu	1.84671587	3.13187344	35.79637464
Cu	0.56872553	4.40986283	37.60372504
Cu	3.12470621	4.40986283	37.60372504
